# New onset diabetes mellitus after kidney transplant: prevalence and risk factors

**DOI:** 10.1186/1758-5996-7-S1-A100

**Published:** 2015-11-11

**Authors:** Júlia Cabral Bastos, Monique Lima e Silva, Giovanna Petrilli, Mariana Arruda Leal Pires, Lívia Carla Ribeiro, Guilherme da Rocha Branco, Layra Faria de Oliveira, Natalia Treistman Frota Leitão, Marcos Miranda Santos Oliveira, Lenita Zajdenverg, Joana Rodrigues Dantas, Melanie Rodacki

**Affiliations:** 1 UFRJ, Rio de Janeiro, Brazil

## Background

New onset diabetes mellitus after transplant (NODAT) has been described in 4-25% of kidney transplant recipients. It is not only a major factor leading to dysfunction and deterioration of the allograft, but also has a significant impact on cardiovascular risk and patient survival. Several risk factors have been linked to this condition such as age, class of immunosupressive drug, obesity and family history of diabetes. However this has been poorly studied in our population.

## Objective

To identify the prevalence and the major risk factors associated with NODAT after kidney transplantation in our population.

## Materials and methods

We performed a retrospective evaluation of patients who underwent kidney transplant from 1994 to 2014 and were not diabetic before the procedure. The prevalence of NODAT was established through the ADA criteria. Clinical and epidemiologic data were retrieved by review of medical charts and analised with SPSS 17.0. A p≤0.05 was considered significant. Results: A total of 109 patients were studied (41,5% female and 58,5% male) Their mean age was 52 (±9.7) yrs. old (range: 27 to 72). Among them, 35 developed NODAT (31,5%). Those who developed NODAT were older than others (mean age 44,9 ±10,1 Vs 40,6 ±10,3; p 0.03). NODAT was more common in those who underwent hemodialysis before the transplant (38,8% Vs 8,3%; p 0.016) and that used imunossupressive therapy with mycophenolate (90,9% vs 73%; p 0.03). BMI before transplantation (p=0.671), gender (p=1.0), ethnicity (p=0,94), type of organ donor (p=0,69), family history of diabetes (p=0,79) and use of other imunossupressive drugs, like tacrolimus (p=0,5), sirolimus (p=0,22), cyclosporine (p=1.0) and corticosteroid (p=0,15), were not associated with NODAT in our patients. The majority of patients using corticosteroids (90,5%) used prednisone dosage ≤ 5 mg/day. In patients who developed NODAT, 2,8% used sulfonylureas alone, 14,3% used metformin alone, 5,7% used DPP4 inhibitors, 2,8% used sulfonylureas and insulin, 17,1% used metformin with insulin and 22,8% used insulin alone.

## Conclusion

NODAT occurs in aproximately one third of patients that underwent kidney transplant in our population. The development of diabetes in these individuals is associated with older age at the time of surgery, hemodialysis before transplant and surprisingly with use of mycophenolate. This finding could be explained by the more common use of this drug in our center than in others.

